# Comparative 454 pyrosequencing of transcripts from two olive genotypes during fruit development

**DOI:** 10.1186/1471-2164-10-399

**Published:** 2009-08-26

**Authors:** Fiammetta Alagna, Nunzio D'Agostino, Laura Torchia, Maurizio Servili, Rosa Rao, Marco Pietrella, Giovanni Giuliano, Maria Luisa Chiusano, Luciana Baldoni, Gaetano Perrotta

**Affiliations:** 1 CNR – Institute of Plant Genetics, Via Madonna Alta 130, 06128 Perugia, Italy; 2 Department of Soil, Plant, Environmental and Animal Production Sciences, University of Naples 'Federico II', Via Università 100, 80055 Portici, Italy; 3 ENEA, TRISAIA Research Center, S.S. 106 Ionica, 75026 Rotondella (Matera), Italy; 4 Department of Economical and Food Science, University of Perugia, Via S. Costanzo, 06126 Perugia, Italy; 5 ENEA, Research Center CASACCIA, S.M. Galeria 00163, Rome, Italy

## Abstract

**Background:**

Despite its primary economic importance, genomic information on olive tree is still lacking. 454 pyrosequencing was used to enrich the very few sequence data currently available for the *Olea europaea *species and to identify genes involved in expression of fruit quality traits.

**Results:**

Fruits of *Coratina*, a widely cultivated variety characterized by a very high phenolic content, and *Tendellone*, an oleuropein-lacking natural variant, were used as starting material for monitoring the transcriptome. Four different cDNA libraries were sequenced, respectively at the beginning and at the end of drupe development. A total of 261,485 reads were obtained, for an output of about 58 Mb. Raw sequence data were processed using a four step pipeline procedure and data were stored in a relational database with a web interface.

**Conclusion:**

Massively parallel sequencing of different fruit cDNA collections has provided large scale information about the structure and putative function of gene transcripts accumulated during fruit development. Comparative transcript profiling allowed the identification of differentially expressed genes with potential relevance in regulating the fruit metabolism and phenolic content during ripening.

## Background

An improvement of our knowledge on gene composition and expression is essential to investigate the molecular basis of fruit ripening and to define the gene pool involved in lipid and phenol metabolism in an oil crop species as olive, characterized by a peculiar fatty acid and antioxidant composition.

The availability of complete genome sequences and large sets of expressed sequence tags (ESTs) from several plants has recently triggered the development of efficient and informative methods for large-scale and genome-wide analysis of genetic variation and gene expression patterns. The ability to monitor simultaneously the expression of a large set of genes is one of the most important objectives of genome sequencing efforts. In this respect, the 454 pyrosequencing technology [1] is a rather novel method for high-throughput DNA sequencing, allowing gene discovery and parallel efficient and quantitative analysis of expression patterns in cells, tissues and organs.

In the past few years, several studies based on comparative high throughput sequencing of plant transcriptomes have, indeed, allowed the identification of new gene functions, contaminant sequences from other organisms, alterations of gene expression in response to genotype, tissue or physiological changes, as well as large scale discovery of SNPs (Single Nucleotide Polymorphisms) in a number of model and non model species, such us maize, grapevine and eucalyptus [2-5].

Olive is the sixth most important oil crop in the world, presently spreading from the Mediterranean region of origin to new production areas, due to the beneficial nutritional properties of olive oil and to its high economic value.

It belongs to the family of *Olea*ceae, order of Lamiales, which includes about 10 families for a total of about 11,000 species. Members of this order are important sources of fragrances, essential oils and phenolics claiming for numerous health benefits, or providing valuable commercial products, such as wood or ornamentals. Information on the genome sequence and transcript profiles of the entire clade are completely lacking.

Olive is a diplod species (2n = 2x = 46), predominantly allogamous, with a genome size of about 1,800 Mb [6,7]. In spite of its economical importance and metabolic peculiarities, very few data are available on gene sequences controlling the main metabolic pathways.

Olive accumulates oil mainly in the drupe mesocarp and its content can reach up to 28–30% of total mesocarp fresh weight. Olive oil shows a peculiar acyl composition, particularly enriched in the monounsaturated fatty acid oleate (C18:1), deriving from the desaturation of stearate. Oleate can reach percentages up to 75–80% of total fatty acids, while linoleate (C18:2), palmitate (C16:0), stearate (C18:0) and linolenate (C18:3) represent minor components. The final acyl composition of olive oil varies enormously among varieties. Environmental factors, such as temperature and light during fruit ripening, can deeply influence the balance between saturated and unsaturated fatty acids [8].

The chemistry of phenolic oleosides is attracting an increasing interest of pharmacological research and agri-food biotechnology, and the biochemical pathway leading to their biosynthesis and regulation has been recently deeply evaluated [9], even if the genetic control still remains completely unknown.

Secoiridoids represent the most important class of phenolics and they arise from simple structures, like tyrosol and hydroxytyrosol, to quantitatively more important conjugated forms like oleuropein, demethyloleuropein, 3-4DHPEA-EDA and ligstroside [10]. Oleuropein is the main secoiridoid, representing up to the 82% of total biophenols, known as the bitter principle of olives and responsible for major effects on human health and for releasing phytoalexins against plant pathogens [10]. Another secoiridoid with relevant health functions is oleocanthal (deacetoxy ligstroside aglycone) [11].

Developing olives contain active chloroplasts capable of photosynthesis, thus representing significant sources of photoassimilates. While chlorophyll is localized mostly in the epicarp, the mesocarp contains significant amounts of other photosynthetic pathway components, such as phosphoenol pyruvate carboxylase [12].

Olive fruit development and ripening, takes place in about 4–5 months and includes the following phases: i) fruit set after fertilization, ii) seed development, iii) pit hardening, iv) mesocarp development and v) ripening. During the ripening process, fruit tissues undergo physiological and biochemical changes that include cell division and expansion, oil accumulation, metabolite storage, softening, phenol degradation, colour change (due to anthocyanin accumulation in outer mesocarp cells). Oil synthesis starts after pit hardening, reaching a plateau after 75–90 days, while the phenolic fraction is maximum at fruit set and decreases rapidly along fruit development.

This work is aimed at defining the transcriptome of olive drupes and at identifying ESTs involved in phenolic and lipid metabolism during fruit development. Drupes from two cultivars have been used: a widely cultivated variety characterized by a very high phenolic content, and an oleuropein-lacking natural variant; two developmental stages, at completed fruit set and at mesocarp development, representing diverse sets of expressed genes, were analyzed using 454 pyrosequencing.

## Results

### Sequencing output

**Figure 1:**
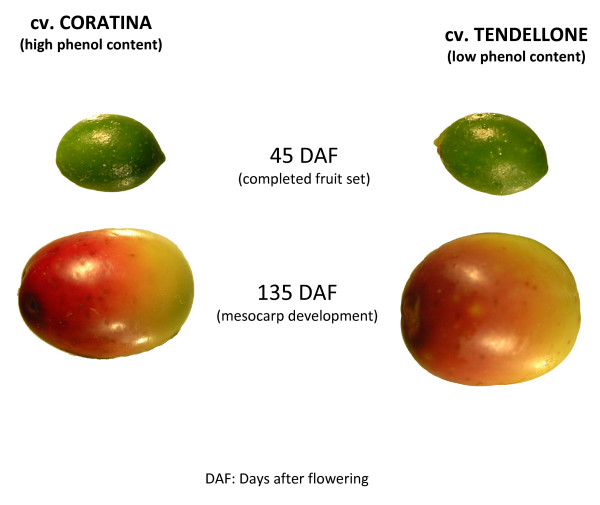
**Plant material**. Fruit mesocarp and epicarp of cvs. *Coratina *and *Tendellone*.

**Figure 2:**
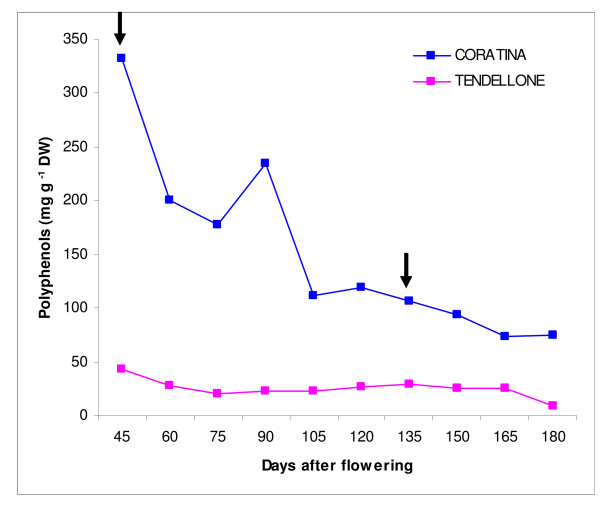
**Changes in polyphenol content between cv. *Coratina *and *Tendellone *in the course of fruit ripening**. Arrows indicate the dates of sample collection: at 45 and 135 DAF.

The starting materials to explore the olive fruit transcriptome were fruit pools from two *Olea europaea *cultivars, *Coratina *and *Tendellone *(C and T), showing striking differences in their biophenol accumulation pattern (Figure 1, 2). C is cultivated in the Apulia region and represents the most widely cultivated variety of Italy, while T is a minor cultivar locally spread in Central Italy. A previous SSR analysis reported a very high genetic distance between them [13]. These cultivars also differ markedly in their oleuropein concentration (272.9 mg g^-1 ^dw in C, decreasing strongly during fruit ripening, and 0.3 mg g^-1 ^dw in T, decreasing only slightly during ripening). In contrast, the content of the 3-4DHPEA-EDA intermediate compound was similar between genotypes (data not shown).

**Figure 3:**
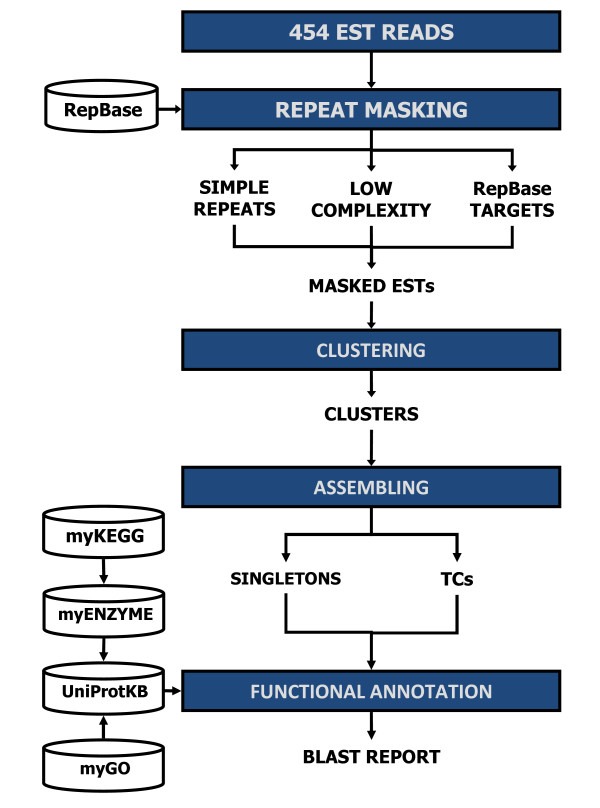
**EST processing work-flow**.

Four enriched full-length ds-cDNA collections (see Methods) were obtained and their 454 pyrosequencing provided a total of 261,485 sequence reads, corresponding to 58.08 Mb, with an average read length of 217–224 nt, depending on the cDNA sample (Table 1). The 4-step procedure adopted by the ParPEST pipeline to process the 454 EST reads is summarised in Figure 3.

**Table 1:** 454 sequencing raw data

**SAMPLE**	**HQ READS**	**HQ BASES**	**AVERAGE LENGTH (bp)**
*Coratina *45 DAF	51,659	11,215,346	217.10
*Coratina *135 DAF	61,488	13,769,788	223.94
*Tendellone *45 DAF	71,112	15,963,353	224.48
*Tendellone *135 DAF	77,226	17,127,266	221.78

	261,485	58,075,754	222.82

HQ READS: Quality passed reads, HQ BASES: Quality passed bases.

ESTs were masked to eliminate sequence regions that would cause incorrect clustering. Targets for masking include simple sequence repeats (SSR, also referred to as microsatellites), low complexity sequences (including poly-A tails) and other DNA repeats. The number of ESTs masked for each category, as well as the total nucleotides that were masked, are shown in Table 2.

**Table 2:** Number of ESTs masked for each mask sequence category

***Cultivar***	*Coratina*		*Tendellone*	
**Developmental stage**	45 Days After Flowering	135 Days After Flowering	45 Days After Flowering	135 Days After Flowering
**Simple repeats**	2,683 (97,992)	3,586 (129,807)	3,168 (113,243)	4,199 (146,670)
				
**Low complexity**	683 (22,961)	779 (23,895)	814 (25,082)	894 (28,066)
**Match in RepBase**	1,781 (29,8882)	2,959 (450,297)	2,663 (468,078)	3,176 (559,319)
	5,147 (419,835)	7,324 (603,999)	6,645 (606,403)	8,269 (734,055)

*The total nucleotides masked is given in brackets

The most frequent DNA repeats, identified using RepBase as the filtering database, were ribosomal RNA (both SSU and LSU); LTR retrotransposons from the BEL type family, Gypsy and Copia; non-LTR retrotransposons from the CR1 superfamily and, finally, a batch of retro pseudogenes (CYCLO, L10, L31, L32) (data not shown).

In order to assess EST redundancy in the whole collection and provide a survey of the *Olea europaea *drupe transcriptome, masked EST sequences were pair-wise compared and grouped into clusters, based on shared sequence similarity. As a consequence, the obtained clusters are ESTs which are most likely products of the same gene. Each cluster was then assembled into one or more tentative consensus sequences (TCs), which were derived from multiple EST alignments. As described in Methods, TCs within a cluster shared at least 90% identity within a window of 100 nucleotides. Therefore, the presence of multiple TCs in a cluster could be due to possible alternative transcripts, to paralogy or to domain sharing. In addition, all the ESTs that, during the clustering/assembling process, did not meet the match criteria to be clustered/assembled with any other EST in the collection, were defined as singleton ESTs. The combination of TCs and singletons are referred to as unique transcripts.

The total number of clusters generated was 22,904. They were assembled into 26,563 TCs comprising 185,913 EST reads. The TC length ranged between 102 (min) and 4,916 (max) nucleotides, while TC average length was 355 nucleotides. 2,406 were the clusters assembled into multiple TCs (ranging from 2 to 14). The total number of singleton ESTs (sESTs) was 75,570, with an average length of 179 nucleotides (Table 3, 4).

**Table 3:** Summary of the EST assembly

**Nr. of cluster**	**Nr. of clusters with multiple TCs**	**Nr. of sESTs**	**Nr. of TCs**	**Nr. of unique transcripts**
22,904	2,406	75,570	26,563	102,133

**Table 4:** Composition of the assembled dataset

***TCs***			
Number of sequences	Average length (nts)	Min seq length	Max seq length
26,563	354.93	102	4,916
			
***ESTs in TCs***			
			
Number of sequences	Average length (nts)	Min seq length	Max seq length

185,913	239.47	101	412
			
***sESTs***			
			
Number of sequences	Average length (nts)	Min seq length	Max seq length

75,570	179.35	36	446

The analysis of the full EST collection from this work revealed an average GC-content of 42.5%, ranging from less than 16% to more than 63%.

### Database web interface

The *OLEA *EST database consists of a main relational database (MySQL) which collects raw as well as processed data generated by ParPEST. This is supported by three local satellite databases: myENZYME, a local copy of the ENZYME repository which was built by parsing the *enzclass.txt *and the *enzyme*.*dat *files (release 04 Nov 2008) retrieved from the ExPASy FTP site; myGO, a mirror of the Gene Ontology database, which was built by running the seqdblite MySQL script (version 20081102) downloaded from the GO database archives; myKEGG, which was built by parsing XML files of the KEGG pathways (release 21 October 2008), retrieved from the KEGG [14] FTP site. A PHP-based web application provides user-friendly data querying, browsing and visualization.

The web interface  includes Java tree-views for easy object navigation as well as the possibility to highlight *on-the-fly *the enzymes in the pathway image files retrieved from the KEGG FTP site.

### Functional annotation

In order to identify *Olea *unigenes coding for proteins with a known function, we used a BlastX-based annotation that provided 12,560 TCs with significant similarities to proteins in the UniProtKB database; the remaining 14,003 (52.7%) had no function assigned. A higher number of sESTs with no function was obtained (58,835), representing 77.85% of the total.

When considering annotated TCs and sESTs with respect to the origin of the protein data source, the bulk of the identifications (73% – 75%), concerned proteins of plant origin, as expected.

**Figure 4:**
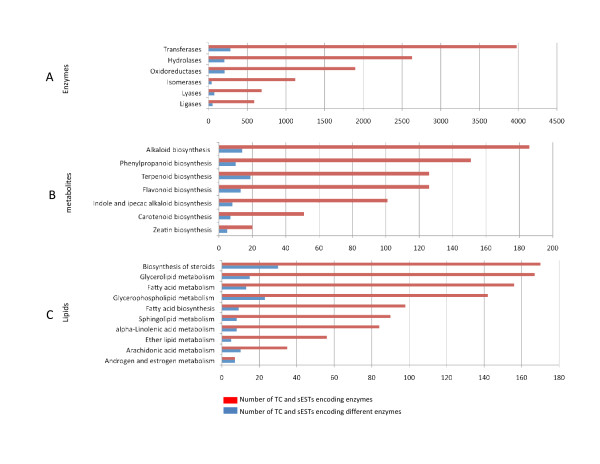
**Depiction of enzyme-encoding TCs grouped by classes, each describing the main enzymatic activity**. (A). B and C represent TCs encoding enzymes involved in biosynthesis of plant metabolites and lipids, respectively. The number of TCs encoding each enzyme class is reported on the x axis of each graph.

TCs and sESTs coding for enzymes with assigned EC number were 5,040 and 5,864, respectively (Figure 4A) following the ENZYME classification scheme . The majority of the enzyme-related unigenes encode for transferases (3,982), hydrolases (2,628) and oxidoreductases (1,895). Of particular relevance for fruit metabolism are those TCs and sESTs involved in the biosynthesis of secondary metabolites (761) and lipids (1,005) (Figure 4B, C). Most frequently, the same enzymatic function is redundantly encoded by several unigenes, this may be the result of different proteins referenced with the same EC number or the effect of different transcripts encoding specific enzyme subunits. Given the limited sequence length typically provided by 454 pyrosequencing, it is also plausible that in some cases different TCs and sESTs cover non-matching fragments of the enzyme transcript coding frame.

### Changes in transcript abundance

In principle, the higher the number of ESTs assembled in a specific TC, the higher the number of mRNA molecules encoding that particular gene in a given tissue sample. However, differences in transcript abundance may reflect sampling errors rather than genuine differences in gene expression. Hence, in order to identify differentially expressed genes in the four sequenced fruit cDNA collections, the statistical R test [15] was applied, as a measure of the extent to which the observed differences in the gene transcription among samples reflect their actual heterogeneity. Applying this test and further filtering criteria to select differentially expressed TCs among the four sets (see Methods), we selected 2,942 differentially expressed TCs, 1,627 of them with a predicted annotation and 1,315 with no similarity with other sequences in the public databases [see Additional file 1].

**Figure 5:**
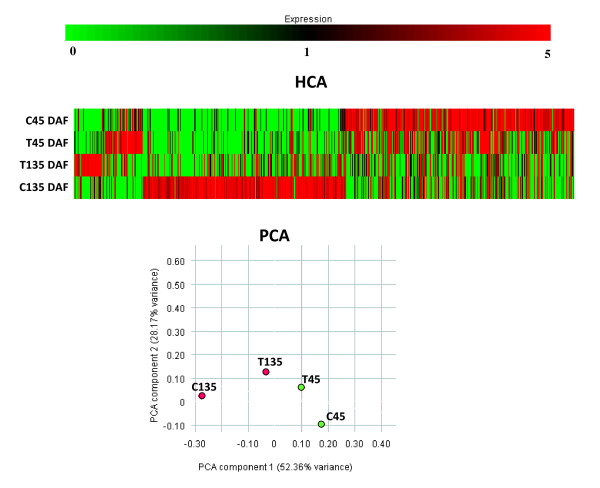
**A. Hierarchical Clustering Analysis (HCA) of differentially expressed TCs**. Color codes for expression values are reported on the top. B – Principal component analysis (PCA) of differentially expressed TCs. The percentage of variance explained by each component is shown within brackets. C45 = *Coratina *45 DAF; C135 = *Coratina *135 DAF; T45 = *Tendellone *45 DAF; T135 = *Tendellone *135 DAF.

Clustering of differentially expressed TCs distinguished gene transcripts differentially expressed during fruit development from those differentially expressed between genotypes, evidencing that the former were more numerous than the latter. C was the genotype showing the highest expression differences between the two stages (Figure 5A). This result was confirmed by PCA analysis, where transcripts from the second stage of C were significantly divergent from the remaining ones (Figure 5B).

**Figure 6:**
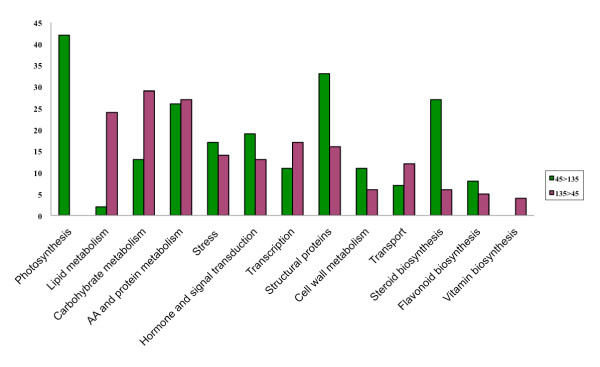
**Transcripts identified as differentially expressed between 45 DAF and 135 DAF in both genotypes grouped in functional categories (listed on the x axis) on the basis of their predicted biological process**.

Transcript differences affect several important physiological processes that promote fruit growth and development. Transcripts identified as differentially expressed between 45 and 135 DAF in both genotypes and between genotypes, grouped in 13 categories on the basis of their predicted annotations, showed that different biological processes are modulated at the molecular level by stringent genetic and developmental signals (Figure 6). Transcripts involved in photosynthesis, in biosynthesis of structural proteins (histones, aquaporins, ribosomal proteins, tubulins, pollen allergens), in terpenoid and flavonoid biosynthesis, in cell wall metabolism, in cellular communication (hormone biosynthesis and regulation, cascades of signal transduction) and responses to biotic and abiotic stresses, were mainly expressed at 45 DAF, whereas the majority of gene transcripts related to different primary metabolic pathways (carbohydrate, lipid, amino acid and protein metabolism) as well as transcription factors and regulators and genes involved in vitamin biosynthesis, were more expressed at 135 DAF (Figure 6).

A wide set of genotype-specific TCs, mainly related to hormone biosynthesis and signalling, responses to abiotic and biotic stresses, biosynthesis of terpenes and phenylpropanoids, were also observed (Table 5).

**Table 5:** TCs assembled from ESTs exclusively present in one of the two cultivars

Biological Process	N. of specific TCs for cv. *Coratina*	N. of specific TCs for cv. *Tendellone*
Hormone metabolism and regulation	13	5
Abiotic and biotic stress	10	4
Cell wall metabolism	9	3
Lipid metabolism	7	7
Steroid metabolism	9	0
Phenylpropanoid metabolism	7	3

**Figure 7:**
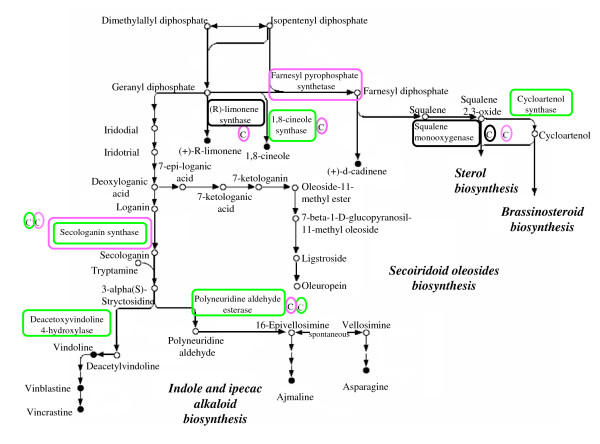
**Partial representation of the metabolic pathway for terpenoid biosynthesis (Kegg map 00900)**. TCs more expressed at 45 DAF are boxed green, while those more expressed at 135 DAF are boxed purple. In black boxes are those TCs expressed at all fruit ripening stages and in both genotypes. Genotype-specific TCs more expressed at 45 DAF, more expressed at 135 DAF and with unchanged expression are included in green, purple and black circles, respectively, and reported with C (*Coratina*) and T (*Tendellone*) when they are exclusively present in one of the two cultivars.

**Figure 8:**
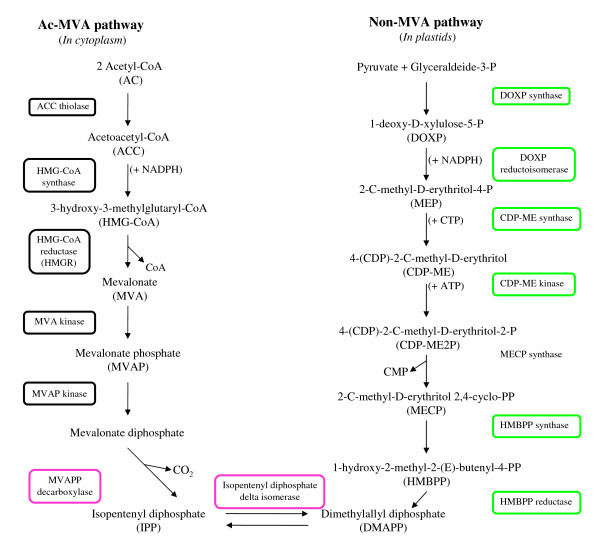
**Partial representation of the metabolic pathway for biosynthesis of steroids (Kegg map 00100)**. Color codes for boxes are the same as in Figure 7.

Furthermore, TCs encoding structural enzymes synthesizing terpenoids and terpenoid precursors (such as dimethylallyl diphosphate (DMAPP) and isopentenyl diphosphate (IPP)) fluctuated between developmental stages (Figures 7 and 8). Transcripts involved in the mevalonate (MVA) pathway for isoprenoid biosynthesis, occurring in the cytoplasm, were predominantly not regulated, while six out of seven genes coding for the main enzymes of the plastidial non-mevalonate (non-MVA) pathway, presented TCs more abundant at 45 DAF.

**Figure 9:**
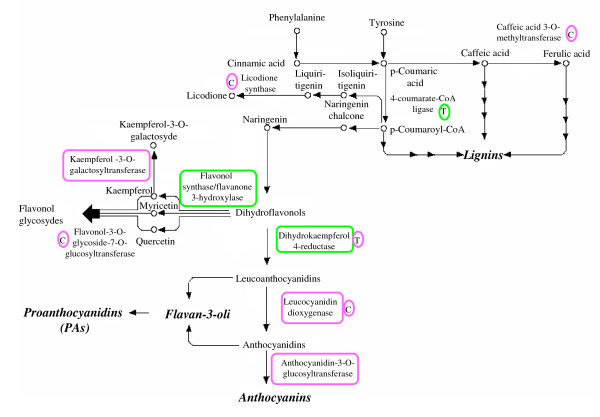
**Partial representation of the metabolic pathways for phenyl-propanoid and flavonoid biosynthesis (Kegg maps 009490 and 00941, respectively)**. Color codes for boxes are the same as in Figure 7.

Finally, transcripts involved in flavonoid biosynthesis were also regulated between developmental stages and genotypes (Figure 9).

## Discussion

### Sequencing output

This is the first report of a large-scale and comparative EST analysis from olive fruit. Olive is one of the most important oil crops in the world. It belongs to the Asterid clade of angiosperms, that includes thousands of economically important crops for which genomic information is still scarce. The massive EST characterization described here can be considered an initial platform for the functional genomics of *Olea europaea *and will be a starting point for the establishment of molecular tools for improving the major quality traits in *Olea *species. Massively parallel EST sequencing provided more than 102,000 unigenes consisting in 26,563 TCs and 75,570 singletons from four fruit libraries. Considering 27 available data on expressed genes of other plant species, such as *Arabidopsis *[16], it is possible that the reported unigene set of *Olea *is an over estimate of the actual number of transcripts expressed in the fruit. This could in part be the result of unassembled segments of TCs and sESTs pertaining to the same transcript unit. A certain amount of incomplete EST assembly is expected as a result of the short reads provided by the 454 pyrosequencing technology.

Despite the fact that cDNA samples were prepared without any normalization process, we only found a moderate degree of redundancy. Clustering of ESTs has indeed reduced the number of sequences by 61% from 261,483 quality passed reads to 26,563 TCs plus 75,570 sESTs. RepBase masking analysis has revealed a surprising amount of short repeats and transposable elements (TEs), which could represent a valuable resource to develop TE-derived molecular markers [17] and to investigate on *Olea *genome size evolution. Also, the GC-content of 42.5%, ranging from less than 16% to more than 63%, can provide a contribution to the evolution studies and gene transfer dynamics within the *Oleaceae *taxon.

### Functional annotation

The percentage of TCs and singletons with no putative function assigned was considerably elevated, possibly as a result of gene functions specifically evolved in *Olea europaea *and quite divergent from those of other plant species. The *Olea *fruit, indeed, presents a number of exclusive traits, like, above all, oil and biophenol accumulation. These traits are encoded at genomic level. On the other hand, the high incidence of unigenes with no assigned function (about 70%), could be due to the poor annotation that still affects protein databases. Also, it is possible that many TCs and sESTs could not be reliably annotated because they did not cover the entire length of the transcript or because they represent untranslated regions (UTRs). This could be particularly the case of our dataset given that the 454 sequencing technology typically provides short sequence reads.

The identification in the *Olea *genome of transcribed sequences similar to a wide range of phylogenetically distant organisms raises intriguing questions about the evolution of their physiological roles and about whether or not these sequences and the related functions are the result of recent gene transfer or the relic of an ancient past.

It is important to note that about 25% of the annotated enzyme-coding transcripts are involved in biosynthesis of lipids and fruit metabolites. The availability of the genetic information related to these enzyme functions represents, in our view, a fundamental tool for understanding the molecular basis of the expression of traits related to fruit phenotype and for establishing new strategies of metabolic engineering to improve the overall quality of olive fruit.

### Changes in transcript abundances

Large scale random sequencing of different fruit cDNA collections has provided information on relative large scale variation of gene expression. However, it should be noted that no further experimental validation has been performed on differentially expressed TCs passing the R test [15].

Analysis of differentially expressed gene transcripts evidenced large differences in key genes involved in a number of metabolic pathways that can potentially alter most quality traits in olive fruits. In some cases, different TCs with identical predicted annotation showed a contrasting accumulation pattern between developing stages or between genotypes; this implies that similar, although not identical, proteins and enzymes may undergo different expression patterns, determining a fine regulation of metabolic pathways and the accumulation of alternative metabolites.

It is interesting to note that the C cultivar underwent a larger degree of transcriptional modulation during fruit development. It is possible that this is related to the very high content in phenolic compounds at the beginning of fruit development in this cultivar.

### Comparison between fruit developmental stages

Expression differences were found for transcripts involved in several physiological processes that promote fruit growth and development. Plant cells require sugars to synthesize lipids and acetyl-CoA is the precursor of carbon chain elongation in all fatty acids. Photosynthesis is an important source of sugars for mesocarp development and olive oil biogenesis. Photoassimilates translocated from leaves to fruit mesocarp by phloem are another indispensable source of sugars in developing fruits [18]. At the end of the ripening process, concurrently to the decrease of chlorophyll content in fruit mesocarp and to the gradual color change, the intense mitochondrial respiration of photoassimilates translocated from leaves to fruits through the phloem, becomes the main energy source sustaining fruit ripening [19]. Consistent with this fact, TCs with predicted functions related to photosynthesis (photoreception, Calvin cycle and oxidative phosphorilation) were more represented at 45 DAF, while transcripts associated with carbohydrate metabolism (glycolysis/gluconeogenesis, citrate cycle, and fructose, mannose and galactose metabolism) were more represented at 135 DAF.

Generally, transcript fluctuations were consistent with the physiological status of the fruit. The higher expression of transcripts related to the biosynthesis of structural proteins at 45 DAF may be correlated with the intense and rapid cell divisions during fruit growth, while the higher expression of transcripts putatively associated with fatty acid biosynthesis and with the assembly of storage triacylglycerols (TAGs) at 135 DAF, is in agreement with fatty acid accumulation pattern in olive fruits, starting at about 90 DAF until the end of fruit maturation [18].

This work has allowed the identification of most TCs related to secoiridoid conjugate (such as oleuropein) biosynthesis, deriving from the conjugation of an esterified phenolic moiety (phenylpropanoid metabolism) and an oleoside moiety (deoxyloganic acid deriving from the mevalonic acid pathway) [9]. TCs related to the mevalonate pathway were not significantly regulated with the exception of MVAPP decarboxylase, leading to the biosynthesis of IPP, a common precursor for all secoiridoid oleosides. Surprisingly, this TC was up-regulated in both cultivars at fruit veraison, when the secoiridoid content is decreasing. The non-MVA pathway, recently discovered in plant chloroplasts, produces various classes of terpenoids, mostly hemiterpenes, monoterpenes, diterpenes and carotenoids [20]. In higher plants, both pathways operate simultaneously and their physical compartmentalization does not preclude exchanges of metabolic intermediates, although the nature of this crosstalk remains to be elucidated [20,21]. It is likely that the specialization of each pathway can play a key role in regulating the biosynthesis of specific end products during olive fruit development.

Given the very high accumulation of secoiridoids (mainly oleuropein) in developing fruits, the terpenoid metabolic pathway must be strongly oriented toward the secoiridoid biosynthesis branch. Unfortunately, the main enzymes and related genes involved in oleuropein biosynthesis are still unknown, hence the information provided by our comparative genomics survey cannot provide direct insight into the molecular basis of secoiridoids accumulation. Since C and T show extremely different oleuropein accumulation patterns, it is likely that transcripts encoding key enzymes for oleuropein biosynthesis show specific differences in their accumulation patterns in these two cultivars. In this respect, mining of TCs differentially expressed in T vs. C, with functional annotations compatible with enzymes for oleuropein biosynthesis, or with no functional annotation, is under way to address this point.

Other classes of phenolic compounds are known to be less represented in olive fruits. Nonetheless, specific alteration of gene transcripts encoding a number of structural enzymes suggests that even metabolites synthesized from secondary branches can show differential expression following developmental and genetic cues.

The common precursor of both secoiridoids and indole alkaloids, is deoxyloganic acid. Also loganin and secologanin, leading to indole alkaloid synthesis, could possibly be involved in biosynthesis of secoiridoids [22]. The fact that three TCs putatively encoding secologanine synthase (EC: 1.3.3.9) are more represented at 45 DAF, while 2 TCs are instead more represented at 145 DAF, deserves further investigation to verify to which extent fine regulation of different secologanine synthases can affect the actual accumulation of specific secoiridoid/indole alkaloid products. Alternate regulation of distinct TCs sharing identical annotations has proven to be a quite common condition in the *Olea *transcriptome [see Additional file 1], suggesting that the well known plasticity of metabolite accumulation in olive fruits could be the result of the fine modulation of genes encoding different enzyme isoforms.

Although the flavonoid content of olive fruits is relatively low compared to other phenolic classes and the pattern of their accumulation during fruit development is still unknown, transcriptional differences observed between 45 DAF and 135 DAF are in general agreement with data available from other fruit species [23].

### Comparison between C and T genotypes

The EST database also contains comparative information between fruits of the C and T genotypes. This can be particularly informative, considering that, as previously reported, the two genotypes have extremely differentiated fruit phenolic content. On the other hand, the lack of genetic information on the biosynthesis of secoiridoids in plants makes it impossible to find orthologs in protein databases, thus precluding the possibility to identify ESTs directly correlated to their accumulation in the olive fruit.

Among genotype-specific transcripts, several TCs putatively involved in the biosynthesis of steroids with nutritional and health benefits, were reported exclusively in C. Two TCs, specific to C, encode R-limonene synthase 1 (EC: 4.2.3.20) and 1,8-cineole synthase (EC: 4.2.3.11), which are related to the biosynthesis of important flavour compounds, such as (+)-R-limonene, one of the most abundant monocyclic monoterpenes in nature [24] and 1,8-cineole, also known as eucalyptol, a monoterpenic oxide present in many plant essential oils.

A number of other genotype-specific TCs could account for biologically relevant differences between C and T and provide a useful hint for focused biochemical analyses. In general, genotype-specific TCs were prevalent in C, supporting the hypothesis that C fruits may synthesize a wider array of secondary metabolites. The *Olea *EST database will be a useful tool for unravelling the biochemical diversity of olive fruits.

## Conclusion

In this work we describe the first large EST collection of *Olea europaea *L. It represents a valuable resource to assist a preliminary evaluation of features from the *Olea europaea *genome (i.e. GC content, SSR, genome annotation). The EST database can be consulted through a user friendly web interface that provides useful tools for data querying, blast services, browsing and visualization.

Comparative sequencing of four fruit cDNA collections has provided information on variation of gene expression during fruit development and between two genotypes with contrasting phenolic accumulation in fruits.

Analysis of differentially expressed gene transcripts evidenced large differences in key genes involved in a number of metabolic pathways that can potentially control most quality traits in olive fruits.

## Methods

### Plant material

Olive drupes of two cultivars were used: *Coratina *(C), a widely cultivated variety, characterized by a very high phenolic content, reaching 332,5 mg/g of total fruit dry weight, and *Tendellone *(T), a low-phenolics natural variant (42,7 mg/g dw). Olive fruits were sampled from plants of the Olive Cultivar Collection held by the CRA-OLI (Collececco, Spoleto). The different olive trees were grown using the same agronomic practices, including irrigation conditions, that can affect phenolic concentration in the fruit. C and T cultivars were selected as the extreme variants in phenolic content among a set of twelve cultivars (including Bianchella, Canino, Dolce d'Andria, Dritta, Frantoio, Leccino, Moraiolo, Nocellara del Belice, Nocellara Etnea, Rosciola) surveyed for two years for oleuropein, demethyloleuropein, and 3–4 DHPEA-EDA content (data not shown). Fruits were harvested at 45 and 135 days after flowering (DAFs). These stages correspond to important physiological phases of fruit development: completed fruit set and mesocarp development, respectively. Only fruit mesocarp and epicarp have been used for RNA extraction.

### cDNA synthesis and 454 sequencing

**Figure 10:**
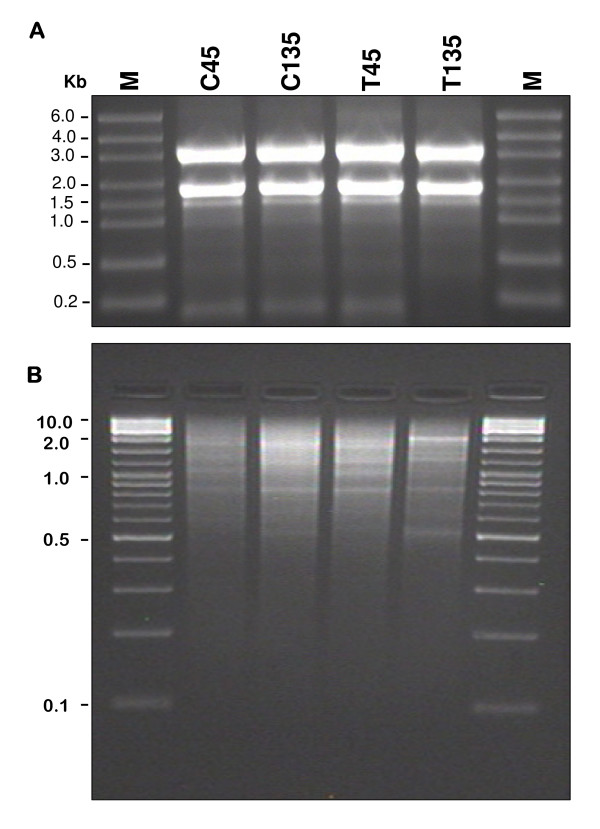
**cDNA sample preparation**. A. Electrophoresis of total RNA on denaturing agarose gel. B. Double stranded cDNA separated on agarose gel electrophoresis. C45 = *Coratina *45 DAF; C135 = *Coratina *135 DAF; T45 = *Tendellone *45 DAF; T135 = *Tendellone *135 DAF; M = Molecular Ladder.

Total RNAs were extracted from pooled fruits using the RNeasy Plant Mini Kit (Qiagen). Contaminating genomic DNA was removed by DNase I (Qiagen) treatment. The RNA was quantified using a spectrophotometer at a wavelength of 260 nm and its quality was checked by running 5 μg of total RNA on 1.2% agarose gel under denaturing conditions (Figure 10A).

The cDNA was prepared by using SMART PCR cDNA Synthesis protocol (Clontech) optimizing the conditions to obtain high quantity of clean cDNA in a small volume. For this reason numerous tests have been performed applying different reaction conditions and purification protocols.

First strand synthesis was performed using 6 ug of total RNA for each sample, in three independent reactions, with the use of Super Script II (Invitrogen) in a reaction mixture containing 50 mM Tris-HCl pH 8.3, 75 mM KCl, 6 mM MgCl_2_, 2 mM DTT. The retro-transcription reaction was primed with 3' SMART CDS Primer IIA (Clontech). The SMART II™ A Oligonucleotide (Clontech), which has an oligo(G) sequence at its 3' end, was used to create an extended template useful for the full-length enrichment provided by SMART™ technology. In fact, when reverse transcriptase (RT) reaches the 5' end of the mRNA, the enzyme's terminal transferase activity adds a few additional nucleotides, primarily deoxycytidine, to the 3' end of the cDNA. The SMART™ II A Oligonucleotide base-pairs with the oligo(G) sequence and RT then switches templates and continues replicating to the end of the oligonucleotide [25]. In cases where RT pauses before the end of the template, the addition of deoxycytidine nucleotides is less efficient than with full-length cDNA-RNA hybrids, thus preventing base-pairing with the SMART™ II A Oligonucleotide. The SMART anchor sequences contained in both 5' and 3' ends of cDNA serve as universal priming sites for end-to-end cDNA amplification. In this manner, SMART method is able to preferentially enrich for full-length cDNAs .

For second strand synthesis, PCR was carried out on a small aliquot (1/10th volume) of the primary template by using Advantage 2 Polymerase Mix (Clontech). The following thermal cycling program was applied: initial denaturation at 95°C for 60 sec, followed by 15 cycles at: 95°C denaturation for 30 sec; 55°C annealing for 30 sec; 68°C extention for 6 min. All the PCR reactions, for each sample, were pooled together and purified by using QIAquick PCR purification kit (Qiagen).

Double stranded cDNA was quantified with a spectrophotometer (NanoDrop 1000, Thermo Scientific) and microplate fluorimeter (Victor 2, Perkin Elmer, Wellesley, MA, USA) and then concentrated by speed vacuum to a concentration of 500 ng/ul. The products were checked on a 2% agarose gel to verify cDNA quality and fragment length. The main size distribution was included between 500 and 4,000 bp (Figure 10B).

Approximately 5 μg of each cDNA sample were sheared via nebulization into small fragments, and sequenced in a single 454 run (the pico-titer plate was divided in four sectors) by using a GS-FLX sequencer (454 Life Sciences, Branford, CT, USA).

Raw unprocessed EST sequences generated from this study have been submitted to Short Read Archive (SRA) division of the Genbank repository. 454 SFF file containing raw sequences and sequence quality information can be access through the SRA web site under accession number SRA008270. Data files can also be directly accessed via FTP .

### Bioinformatics EST processing protocol

ESTs were processed using the ParPEST (Parallel Processing of ESTs) pipeline [26] that has been tweaked in order to properly manage shorter length error prone sequences, such as 454 EST reads.

Masking of simple sequence repeats (SSR) and low complexity sub-sequences was performed using the RepeatMasker tool . In addition, EST reads were screened against RepBase (version 13.06) [27], a library of repetitive elements, and returned as masked sequences ready for the clustering/assembling and annotation procedures. This process produced a set of unique transcripts divided into singleton ESTs (sESTs) and tentative consensus sequences (TCs), grouped by clusters. Each cluster comprises TCs sharing at least 90% of identity within a 100 nucleotide window.

BLAST similarity searches (*e-value *1e10^-3^) against the UniProtKB database (version 13.3) [28] were performed to assign a biological function to the unique transcripts.

In addition, Gene Ontology (GO) terms [29] and Enzyme Commission (EC) numbers [30] were associated to each transcript via the UniProtKB accession, and used for the description of sequence function.

To assess the relative abundance of gene transcripts among cDNA samples we applied the statistical R test [15]. All TCs with R>8 (true positive rate of ~98%) and with a minimum 3-fold EST number difference in at least one sample out of the four sequence sets, were considered as differentially expressed.

Hierarchical clustering analysis (HCA) and principal component analysis (PCA) of the data were performed using GeneSpring version 7.3 (Agilent, Santa Clara, CA, USA).

## Authors' contributions

FA carried out RNA extractions and cDNA synthesis. ND performed sequence alignment, assembling, annotation and database construction. LT participated in data mining and design of some figures. MS provided samples and contributed in biochemical inferences. RR supervised the work of FA. MP participated in cDNA synthesis. GG participated in design, drafting and editing of the manuscript. MLC supervised data analysis process and database construction. LB conceived of the study, and participated in its design and coordination. GP conceived of the study, and participated in its design and coordination.

All authors read and approved the final manuscript.

## Supplementary Material

Additional file 1 **List of TCs**. the data provided include the list of TCs. For each TC, ESTs contribution per genotype and per developing stage is also provided. Click here for file
